# Aortoileal fistula following endovascular repair of an abdominal aortic aneurysm: a case report

**DOI:** 10.3389/fcvm.2026.1840464

**Published:** 2026-07-22

**Authors:** Yanfei Feng, Yifeng Pan, Hanlei Zhou, Xiangkun Meng, Juejue Wang, Bing Chen

**Affiliations:** Department of Vascular Surgery, the Second Affiliated Hospital, Zhejiang University School of Medicine, Hangzhou, Zhejiang Province, China

**Keywords:** abdominal aortic aneurysm, aortoenteric fistula, aortoileal fistula, case report, endovascular abdominal aortic aneurysm repair

## Abstract

**Introduction:**

Aortoenteric fistula (AEF) following endovascular aortic repair (EVAR) for abdominal aortic aneurysm (AAA) is a rare but severe complication, with most reported cases involving the duodenum, and involvement of the ileum is exceptionally uncommon.

**Case presentation:**

We report a rare case of secondary aortoileal fistula with concomitant graft infection in a 66-year-old male, occurring two years after EVAR for AAA. The follow-up imaging 2 years after EVAR showed no aneurysmal sac regression, suggestive of a type II endoleak, which was treated with coil embolization. One week post-embolization, the patient presented with abdominal pain and fever, and a secondary aortoileal fistula was diagnosed. The patient underwent bilateral axillofemoral bypass, excision of the AAA and endograft, and intestinal perforation repair. Intraoperative cultures grew Lactobacillus paracasei. The patient recovered uneventfully and remained well at 1-year follow-up.

**Conclusion:**

Aortoileal fistula after EVAR for AAA is an extremely rare yet potentially fatal complication and should be recognized by both clinicians and patients. It may occur as early as 1 month post-operatively or even more than 10 years after EVAR. Appropriate surgical management should be considered on an individual basis to achieve favorable outcomes.

## 1 Introduction

Aortoenteric fistula (AEF) is a rare but life-threatening complication and can be classified as primary or secondary AEF. Secondary AEF denotes an AEF that develops after surgical repair of an aortic aneurysm. Most secondary AEFs occur after open abdominal aortic reconstruction (1). AEF following endovascular abdominal aortic aneurysm repair (EVAR) is quite uncommon, with an incidence ranging from approximately 0.26%–3.66% (2–4). The vast majority of fistulas involve the duodenum, whereas those affecting other segments of the small intestine or the colon are exceedingly rare (5). The reported in-hospital mortality rate for patients who developed AEF after EVAR is 22% (4), while the perioperative mortality for those treated with endovascular repair is approximately 37% (6). However, mortality approaches 100% in patients who do not undergo timely or feasible surgical intervention (7, 8).

**Figure 1:**
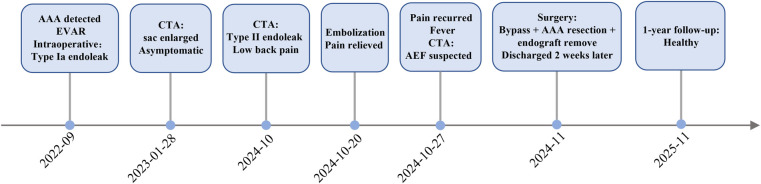
Timeline of the case report.

We report the case of a 66-year-old man who underwent EVAR for an abdominal aortic aneurysm (AAA) and subsequently developed an aortoileal fistula 2 years after the procedure. The fistula occurred in the ileum rather than the more commonly involved duodenum. The patient was treated with axillo-bifemoral bypass, graft explantation, and ileal repair and remained alive at the 1-year follow-up (Figure 1).

## 2 Case description

**Figure 2:**
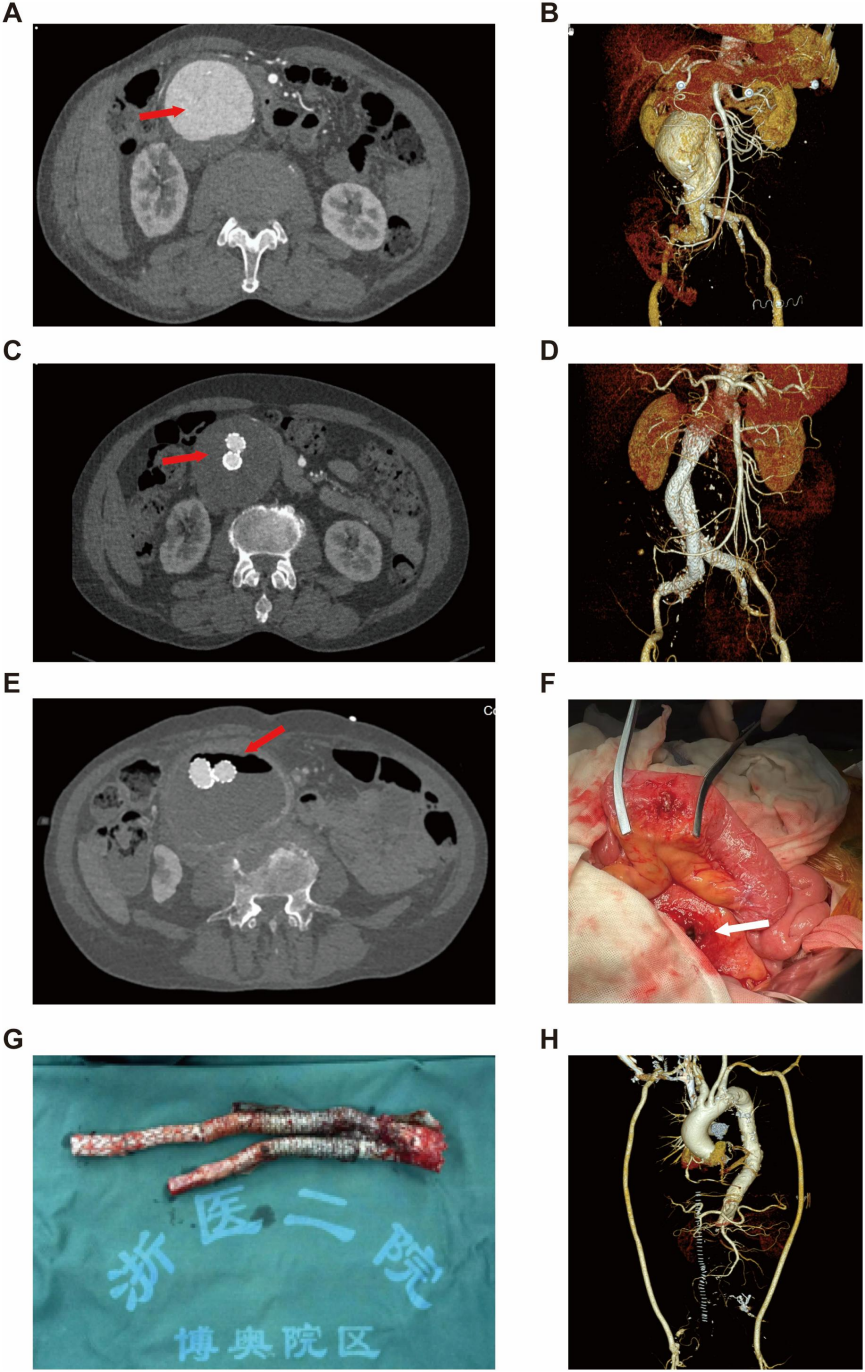
Imaging and intraoperative findings of the case report. **(A)** Preoperative CTA on September 12, 2022, demonstrating an infrarenal abdominal aortic aneurysm with a maximum diameter of approximately 77 mm (red arrow). **(B)** Three-dimensional (3D) reconstruction of the same preoperative CTA, clearly showing the overall morphology, tortuosity, and extent of the infrarenal abdominal aortic aneurysm. **(C)** Follow-up CTA on January 28, 2023, showing the stent graft in place within the abdominal aorta and bilateral common iliac arteries, with a circumferential low-attenuation rim around the graft aneurysmal dilatation of the sac with a maximum diameter of approximately 91 mm (red arrow). **(D)** 3D reconstruction of the same follow-up CTA corresponding to panel C. **(E)** CTA on January 15, 2025, demonstrating postoperative changes following EVAR with covered stent graft and iliac artery balloon angioplasty, with multiple gas shadows visible within the original aneurysmal sac (red arrow). **(F)** Intraoperative photograph during exploratory laparotomy, revealing an ulcerative lesion of the ileum approximately 260 cm distal to the ligament of Treitz, adherent to the abdominal aortic lesion (white arrow). **(G)** Photograph of the explanted endovascular stent graft removed during reoperation. **(H)** 3D reconstruction of CTA performed one week post-operatively.

On September 12, 2022, a 66-year-old male was found to have an AAA during a health check-up, with no significant presenting symptoms or complaints. Abdominal computed tomography angiography (CTA) showed aneurysmal dilatation of the infrarenal abdominal aortic lumen with a maximum diameter of approximately 77 mm (Figures 2A,B**)**. He subsequently underwent EVAR using a bifurcated covered stent graft (main body: Gore Excluder, 28 × 14 × 180 mm; iliac limbs: Gore, 16 × 12 × 140 mm, 16 × 12 × 180 mm, and 16 × 14 × 100 mm), bilateral common iliac artery stent implantation, and embolization of the right internal iliac artery with coils (Cook, sizes 10 mm, 16 mm, and 4 mm). Intraoperative angiography demonstrated a proximal type Ia endoleak; despite balloon dilation at the graft junction, a small amount of proximal endoleak persisted, while both iliac arteries opacified well. Postoperative surveillance imaging was therefore planned and conducted at regular intervals.

On January 28, 2023, CTA demonstrated a circumferential low-attenuation rim around the stent graft, with a maximum diameter of approximately 91 mm **(**Figures 2C,D**)**. The aneurysm sac appeared slightly larger than before surgery, but no obvious contrast filling was observed. The patient had no significant complaints, so continued close observation was recommended.

In October 2024, the patient developed low back pain without fever, chills, or other discomfort. CTA showed enlargement of the aneurysm sac to a diameter of 11 cm. Angiography suggested localized accumulation of contrast medium around the graft, suggestive of a possible type II endoleak (external hospital data unavailable for provision). On December 20, 2024, he underwent embolization of an internal iliac artery branch endoleak, after which his back pain was relieved. One week later, the patient again developed low back pain accompanied by fever (maximum temperature 38.2 °C) and was subsequently transferred to our hospital for further management.

After transmission, CTA showed postoperative changes following EVAR with covered stent graft and iliac artery balloon angioplasty, with multiple gas shadows within the original aneurysmal sac **(**Figure 2E**)**, raising concern for an internal fistula with the small intestine. Laboratory tests showed elevated C-reactive protein (CRP; 184 mg/L), interleukin-6 (IL-6, 232 pg/mL), and erythrocyte sedimentation rate, with mild anemia (hemoglobin, 93 g/L), positive fecal occult blood testing, and an elevated D-dimer level. Blood cultures were negative for bacteria and fungi. Combining all the examinations, secondary AEF and graft infection were highly suspected. Surgical intervention was therefore undertaken. First, bilateral axillofemoral bypass using prosthetic grafts was performed. Upon completion, exploratory laparotomy revealed an ulcerative lesion of the ileum approximately 260 cm distal to the ligament of Treitz, adherent to the abdominal aortic lesion **(**Figure 2F**)**. The AAA measured approximately 8 cm × 8 cm; the lumen was rigid, with no obvious pulsation observed. The aneurysm was then excised, and the endograft was removed **(**Figure 2G).

Postoperatively, the patient was transferred to the intensive care unit. He was extubated on postoperative day 1 and, with stable vital signs, was transferred to the general ward. Intraoperative cultures of infected tissue grew *Lactobacillus paracasei*. Based on susceptibility testing, vancomycin and linezolid were administered, and anticoagulation with warfarin was initiated. CTA of the entire aorta at discharge showed patent bypass grafts and no evidence of leakage from the residual stumps of the abdominal aorta and bilateral iliac arteries **(**Figure 2H**)**. Concomitant pyogenic osteomyelitis was diagnosed; orthopedic consultation recommended continuation of antimicrobial therapy and lumbar immobilization with a brace. The patient was discharged in good condition 2 weeks after surgery. At the 12-month follow-up, the patient had a favorable outcome.

## 3 Discussion

AEF after EVAR is rare (incidence 0.26%–3.66%) but life-threatening. Although the duodenum is the most commonly involved site, fistulas affecting the jejunum (9), ileum, or colon (10) are exceedingly rare. To our knowledge, this is the first reported case of aortoileal fistula after EVAR, bringing the total number of cases with detailed patient-level data in the English literature to 43 (Table 1). These cases demonstrate marked male predominance (90.48%), with a mean age of 71.7 years and a median interval from EVAR to AEF of 18 months (range 1–180 months) (Table 2).

**Table 1:** Aorto-enteric fistulas after EVAR in English literature.

Date	Author	Age/Sex	Clinical presentation	Period^a^ (months)	Treatment for secondary AEF	Outcome	Estimated etiology
2026	Prent	66/M	Back pain, fever	27	Endograft explant, extra-anatomic revascularization, bowel repair	Alive at 12 months	Endoleak
2025	Romanowska et al.	68/M	Melena and fatigue	92	Abdominal drainage and antibiotic	Died	Endoleak
2023	Oka et al.	86/M	Malaise and melena	12	Endograft explant, *in situ* reconstruction and bowel repair	Died after 4 weeks	IgG4-related periaortitis
2022	White et al.	69/M	Back pain, melena, fever	36	Endograft explant, extra-anatomic revascularization, bowel repair	Alive at 3 months	Unknow
2022	Li et al.	71/M	Hematemesis, melena, fever	6	Endograft explant, *in situ* reconstruction and bowel repair	Alive at 6 months	Unknow
2021	Moriyama et al.	70/M	Fever	24	Endograft explant, *in situ* reconstruction and bowel repair	Alive at 35 months	Unknow
2021	Joshi et al.	80/M	Fever, chills and melena	2	Only bowel repair	Alive at 9 months	Unknow
2021	Gunawardena et al.	73/	Melena, fever	39	Endograft explant, extra-anatomic revascularization, bowel repair	Died after 12 days	Type II endoleak.
2021	Hassan et al.	65/M	Hemorrhage of digestive tract	24	Endograft explant, *in situ* reconstruction and bowel repair	Alive at 2 months	Unknow
2020	Nguyen et al.	85/F	Abdominal pain	25	Endograft explant, extra-anatomic revascularization, bowel repair	Alive at 24 months	Unknow
2020	Hosaka et al.	79/M	Fever and melena	15	Endograft explant, *in situ* reconstruction and bowel repair	Died after 25 months	Unknow
2020	Hosaka et al.	71/M	Back pain	58	Endograft explant, *in situ* reconstruction and bowel repair	Alive at 69 months	Type II endoleak
2020	Hosaka et al.	76/M	Anorexia and back pain	74	Endograft explant, *in situ* reconstruction and bowel repair	Alive at 53 months	Type II endoleak
2020	Hosaka et al.	81/M	Chest pain and melena	15	Endograft explant, *in situ* reconstruction and bowel repair	Alive at 46 months	Type II endoleak
2019	Walter et al.	75/M	Fever, abdominal pain	48	Endograft explant, *in situ* reconstruction and bowel repair	Alive at 5 months	Unknow
2018	Wang et al.	87/M	Melena, fevers	20	Abdominal drainage and antibiotic	Alive at 23 months	Unknow
2018	Arworn et al.	43/M	Hematemesis and melaena	13	Endograft explant, extra-anatomic revascularization, bowel repair	Alive at 9 months	Behcet's disease
2018	Jiang et al.	85/M	Melena, tiredness, fever	30	Endograft explant, extra-anatomic revascularization, bowel repair	Alive at 15 months	Unknow
2016	Gülcü et al.	72/M	Septic syndrome	14	Endograft explant, extra-anatomic revascularization, bowel repair	Unclear	Fungal infection
2016	Kadhim et al.	66/M	Confusion, fever	180	Bowel repair	Alive at 12 months	Infection
2014	Zaki et al.	75/M	Abdominal pain, hematemesis	6	Intraoperative death	Intraoperative death	Unknow
2014	Sörelius et al.	58/M	Abdominal pain, fever	30	Endograft explant, extra-anatomic revascularization, bowel repair	Alive at 48 months	Stent graft had kinked
2014	Kasashima et al.	79/M	Abdominal pain, melaena	4	None	Died	IgG4-related periaortitis
2013	Zhang et al.	65/M	Fever	38	Endograft explant, extra-anatomic revascularization, bowel repair	Alive at 1 months	Unknow
2012	Veraldi et al.	71/M	Melaena, lipothymia	72	Endograft explant, *in situ* reconstruction and bowel repair	Alive at 12 months	Unknow
2012	Kao et al.	82/M	Bloody diarrhea	3	Endograft explant, extra-anatomic revascularization, bowel repair	Died after 2 days	Unknow
2011	McPhee et al.	88/F	Nausea, dizziness	48	Endograft explant, extra-anatomic revascularization, bowel repair	Alive at 5 months	Type II endoleak
2009	Tromp et al.	68/M	Symptom free	1	Endograft explant, *in situ* reconstruction and bowel repair	Alive at 6 months	Endoleak
2009	Lane et al.	69/M	Fever, chills, lethargy, diarrhea	6	Endograft explant, extra-anatomic revascularization, bowel repair	Alive at 2 weeks	Unknow
2009	Chenu et al.	67/M	Fever and lumbar pain	14	Endograft explant, *in situ* reconstruction and bowel repair	Alive at 2 months	Unknow
2007	Ruby et al.	80/M	Abdominal pain, nausea	58	Endograft explant, *in situ* reconstruction and bowel repair	Alive at 13 months	Unknow
2006	Ghosh et al.	52/M	Abdominal and back pain, hemat-emesis	9	None	Died after 1 days	Stent-graft infection
2004	French et al.	69/F	Digestive hemorrhage	16	Endograft explant, extra-anatomic revascularization, bowel repair	Died at 6 days	Stent-graft infection
2003	AbouZamzam et al.	67/M	Abdominal pain	11	Endograft explant, extra-anatomic revascularization, bowel repair	Alive at 4 months	Unknown endotension?
2003	Bertges et al.	78/M	Infection, vomiting	53	Endograft explant, extra-anatomic revascularization, bowel repair	Alive at 1 month	Endoleak coil
2003	Elkouri et al.	78/F	Hematemesis and melena	17	Endograft explant, extra-anatomic revascularization, bowel repair	Died at 12 h	Endoleak coil
2003	Alankar et al.	76/M	Abdominal pain, hematochezia	4	Endograft explant, *in situ* reconstruction and bowel repair	Alive at 6 months	Type I endovascular leak
2002	Kar et al.	78/M	Fever, malaise	20	Endograft explant, *in situ* reconstruction and bowel repair	Alive at 1 year	Unknown endotension?
2001	Parry et al.	61/M	Digestive hemorrhage	6	Endograft explant, *in situ* reconstruction and bowel repair	Alive at 7 months	Peri-aortic inflammatory mass
2000	Makar et al.	70/M	Abdominal pain, fever, digestive hemorrhage	4	Antibiotic only	Died	Crohn's disease
2000	Janne et al.	62/M	Digestive hemorrhage, infection	22	Endograft explant, extra-anatomic revascularization, bowel repair	Alive at 40 months	Migrated and kinked stent graft
1999	Hausegger et al.	53/M	Abdominal pain, digestive hemorrhage	18	Endograft explant, *in situ* reconstruction and bowel repair	Alive at 6 months	Migrated and kinked stent graft
1998	Norgren et al.	71/M	Abdominal pain, digestive hemorrhage	17	Endograft explant, *in situ* reconstruction and bowel repair	Alive at 6 months	Peri-aortic inflammatory mass and ruptured graft

a Period: Time after EVAR; F, female; M, male.

**Table 2:** Summary of data from all 43 cases of AEF after EVAR in the English literature.

Characteristics	n (%) or mean ± SD (range)
Age (year) (*n* = 43)	
Mean	71.7 ± 9.62 (43–88)
Sex (male/female), (male %) (*n* = 42)	38/4 (90.48%)
Location of intestinal fistula (*n* = 43)	
Duodenum	40 (93.02%)
Jejunum	1 (2.33%)
Ileum	1 (2.33%)
Colon	1 (2.33%)
Time after EVAR (months)	28.63 ± 32.00 (1–180)
Vascular reconstruction (*n* = 36)	
In situ reconstruction	19 (52.78%)
Extra-anatomic revascularization	17 (47.22%)
Overall mortality rate (*n* = 42)	11/42 (26.19%)
Mortality rate of surgical patients	6/36 (16.67%)
Mortality rate of nonsurgical patients	4/5 (80.00%)
Number of intraoperative deaths	1

The pathogenesis of post-EVAR AEF is multifactorial. Established mechanisms include mechanical erosion by the stent graft, endograft migration or kinking (11), barbs/hooks injury (12), and persistent aneurysm sac pressurization due to endoleak or endotension (13). Type II endoleak is the most frequently reported cause of secondary enteric fistulization after EVAR (14). Additionally, coils left *in situ* after endoleak embolization may cause delayed bowel erosion (15). Other rare contributing factors include IgG4-related periaortitis and underlying inflammatory bowel disease (16). In the present case, a type II endoleak was identified and treated with coil embolization, followed by symptom onset only 7 days later. In contrast to the typically delayed presentations (months to years), this short interval raises the possibility that the embolization procedure itself may have accelerated fistula formation through local mechanical irritation, coil migration, or induction of inflammation. Rapid sac enlargement (from 9.1 cm to 11 cm) also occurred prior to fistula diagnosis. We hypothesize that coil embolization, particularly when followed by rapid sac enlargement, may convert a low-grade chronic inflammatory state into an acute fistulogenic process through localized ischemia, foreign body reaction, or micro-perforation, beyond simple mechanical erosion.

The ileal involvement is noteworthy, as the ileum is mobile and usually distant from the graft. Intraoperative adherence between the ileal lesion and aortic sac suggests that periaortic adhesions combined with chronic inflammation and sac expansion enabled fistula formation at this unexpected site.

Clinically, AEF presents with nonspecific symptoms. In this patient, recurrent low back pain and fever were prominent, associated with vertebral osteomyelitis. Key early warning signs include rapid sac enlargement, persistent back pain, perigraft gas/fluid on CT, and elevated inflammatory markers. These should prompt urgent evaluation for graft infection or AEF. Differential diagnoses include type II/III endoleak, endotension, graft infection, and post-interventional inflammation. Contrast-enhanced CT is the first-line imaging modality.

Management remains challenging. In the 43-case analysis, overall mortality was 26.19% (11/42) (17), with markedly higher rates in non-operatively managed patients (60% in-hospital) (18, 19). The 2016 AHA scientific statement recommends extra-anatomic revascularization for typical cases, while *in situ* reconstruction may be considered in selected low-risk patients (20). Among the 43 post-EVAR AEF cases, 36 underwent open surgery: 17 had extra-anatomic revascularization (e.g., axillofemoral bypass with graft excision and debridement), which eradicates infection but risks stump blowout, longer operative time, and greater trauma. The other 19 had *in situ* reconstruction, which is more anatomical, avoids stump rupture and additional incisions, and lowers amputation risk (21). Conduits include biologic or nonbiologic materials (22, 23), and omental flap coverage may facilitate infection control (24). No significant differences in mortality or infection-related complications have been shown between the two approaches (25), but *in situ* reconstruction should be avoided with extensive purulent peritoneal contamination. In emergencies (e.g., hemodynamic instability, massive hemorrhage, or poor surgical tolerance), endovascular therapy can serve as a temporizing measure (5), as one retrospective study found better perioperative survival with stent-grafts for life-threatening bleeding than with open surgery (5). In the present case, the patient underwent bilateral axillofemoral bypass, graft explantation, and primary ileal repair. For ileal fistulas, intestinal repair is generally straightforward compared with duodenal fistulas.

Culture-directed antibiotic therapy is mandatory. Intraoperative tissue yielded Lactobacillus paracasei; although often considered a commensal, its isolation in pure culture from periaortic tissue in the context of enteric fistula supports its role as a true enteric pathogen. We recommend at least 6–8 weeks of intravenous antibiotics followed by oral therapy, tailored to clinical response. Lifelong suppression may be required in cases of incomplete graft removal or highly virulent organisms (14).

This case provides several actionable insights for clinical practice. First, recent coil embolization for type II endoleak warrants close surveillance for accelerated AEF, particularly if new back pain or rapid sac enlargement develops. Second, sac enlargement and recurrent back pain are critical warning symptoms that should prompt early CTA or endoscopy. Third, AEF may involve mobile bowel segments such as the ileum when periaortic adhesions are present, emphasizing the need for thorough imaging and intraoperative inspection of all adherent bowel loops. Finally, timely extra-anatomic revascularization combined with graft removal can achieve favorable outcomes when fistula location allows simpler bowel repair.

## 4 Conclusion

Aortoileal fistula after EVAR for AAA is an extremely rare yet potentially fatal complication and should be recognized by both clinicians and patients. It may occur as early as 1 month post-operatively or even more than 10 years after EVAR; therefore, lifelong follow-up is needed. Once the diagnosis is established, in addition to antibiotic therapy, appropriate surgical management should be considered on an individual basis to achieve favorable outcomes.

## 5 Limitations

This study has several limitations. As a single case report, the findings may not be generalizable to broader patient populations. The precise mechanism of aortoileal fistula formation in this patient remains speculative due to the complex interplay of potential contributing factors, including type II endoleak, coil erosion, and possible inflammatory processes. Additionally, long-term outcomes beyond one year of follow-up are not available, limiting assessment of the durability of the surgical treatment.

## 6 Highlights

1. AEF following EVAR for AAA is a rare but severe complication.
2. Most reported AEF cases involved the duodenum.
3. Aortoileal fistula is exceptionally uncommon.
4. We report a case of aortoileal fistula with successful surgical management.
5. Lifelong follow-up is necessary to AEF patients.

## Data Availability

The raw data supporting this case report are available from the corresponding author upon reasonable request.
